# Icariin, A Novel Promising Complementary Therapeutic Strategy in the Management of Female Infertility: A Literature Review -

**DOI:** 10.31661/gmj.v12i.2528

**Published:** 2023-09-12

**Authors:** Sima Mosavi, Amirabbas Rostami, Marzieh Pooladi, Mahdie ShojaeiBaghini, Sahar Poudineh, Maryam Poudineh, Esmaeil Behzadi

**Affiliations:** ^1^ Department of Obstetrics and Gynecology, School of medicine, Urmia university of medical sciences, Urmia, Iran; ^2^ Department of Internal Medicine, Faculty of General Medicine, Yerevan State Medical University aer Mkhitar Heratsi, Yerevan, Armenia; ^3^ Department of Anatomical Sciences, School Of Medicine, Isfahan University of Medical Sciences, Isfahan, Iran; ^4^ Medical Informatics Research Center, Institute for Futures Studies in Health, Kerman University of Medical Sciences, Kerman, Iran; ^5^ School of Medicine, Mashhad Azad University, Mashhad, Iran; ^6^ School of Medicine, Shiraz University of Medical Sciences, Shiraz, Iran

**Keywords:** Icariin, Female Infertility, Female Reproductive System

## Abstract

The prevalence of pre-menopausal female infertility is increasing considerably due to various causes such as environmental pollutants, increased administration of chemotherapeutics and radiation exposure, microbial infections, and genetic/epigenetic alterations. However, the current therapeutical strategies remain unfavorite as the disadvantages are strongly challenging. Icariin (ICI) is a phytoestrogen that exerts some promising properties in order to alleviate female infertility. Therefore, the current literature review aimed to evaluate the conducted studies regarding the beneficial impacts of ICI on the female reproductive system and female fecundity. The findings of the present study revealed that ICI is able to modulate the levels of reproductive hormones as it causes a significant decrement in the levels of luteinizing hormone (LH) and follicle-stimulating hormone (FSH) while increasing the levels of estrogen and progesterone. Furthermore, the administration of ICI results in a dramatic alteration in the expression of sex steroidsâ€™ receptors, particularly in female reproductive tissues. In addition, preserving ovarian follicular reserve, improving the ovarian and uterine histoarchitecture, elongating the estrous cycle duration, and eventually advancing the female fecundity are other major effects of ICI on the female reproductive system. Despite these desired beneficial properties, the current knowledge appears to be insufficient, hence further investigations, particularly on humans, are encouraged. To the best of our knowledge, this review provides a comprehensive information regarding the beneficial effects of Icariin on female infertility for the firs time

## Introduction

nfertility, an integral part of reproductive health which is become a priority global health issue, is defined by the world health organization as a failure of a couple to conceive a known pregnancy after twelve months or longer of regular unprotected sexual intercourse [1][2][3]. 

It is documented that approximately 30 percent of couples in western countries are suffering from infertility. 

Females exert a more critical role in the prevalence of infertility since 20-70 percent of infertility-related factors are associated with women [4][5]. 

A number of factors could cause female infertility including reproductive system disorders (e.g. endometriosis and polycystic ovary syndrome) [4][6], toxicants and environmental pollutants (e.g. organophosphates and carbamates) [7][8], chemotherapies and pharmaceutics (doxorubicin and irinotecan) [9][10], radiation, etc. [11]. 

The current therapeutic strategies include sex hormone therapy such as follicle-stimulating hormone (FSH) therapy, human chorionic gonadotropin, estrogen replacement therapy, etc., assisted reproductive technology, and tubal plastic surgery which despite some desired advances, these strategies are invasive, expensive, and are faced with unavoidable adverse effects like increasing the risk of ovarian hyperstimulation syndrome (OHSS), mental disorders, and so on [1][12][13]. 

Regarding the two principal aspects of female infertility, one of which is the misregulation of sex hormone levels, especially estrogen, and the other is the involvement of oxidative stress [14][15], researchers have been efforting in recent years to find an ideal phytoestrogen to support the ability of couples to re-create. 

**Figure 1. Chemical structure of icariin:**
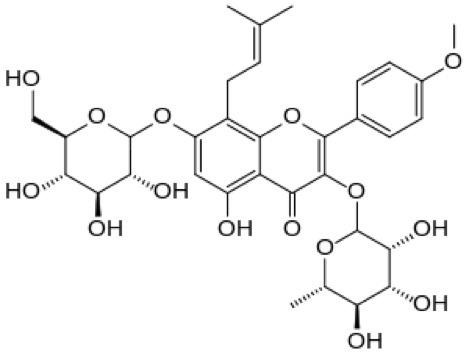


Icariin (ICI) (2-(4′-methoxylphenyl)-3-rhamnosido-5-hydroxyl-7-glucosido-8-(3′-methyl-2-butylenyl))-4-chromanone) is a well-known phytoestrogen extracted from Epimedium pubescens. Chemical structure of ICI is shown in Figure-1. 

This pentenylated flavonoid glycoside monomer exerts a number of desired biological properties such as antioxidant, anti-inflammatory, and anti-tumor activities. 

Therefore, some recent studies have investigated its therapeutic effects on human disorders such as cancer, asthma, cardiovascular disease, osteoporosis, etc. [16][17][18][19]. 

Regarding the documented experimental studies that revealed the beneficial effects of ICI on the female reproductive system, the current literature review aimed to assess the potential therapeutic properties of ICI on female infertility by considering the effects it has on the biosynthesis of reproductive hormones, the structure of uterine and ovarian tissues, the expression of sex steroid receptors, and fecundity. The search was performed in Web of Science, PubMed, Scopus, and Embase databases without time limitation.

Figure-1. Chemical structure of icariin.

## Icariin Modulates Reproductive Hormones Synthesis 

The hypothalamic-pituitary-ovarian (HPO) axis, a highly regulated system, controls 

female reproduction through cyclic biosynthesis of gonadotropic and steroid hormones [20]. 

Due to the vital role of hormones secreted by the HPO axis in preparing reproductive tissues for healthy reproduction, any dysregulation will result in impaired reproductive function, reproductive-related disorders such as premature ovarian failure (POF), polycystic ovarian syndrome (PCOS), ovarian and uterine cancer, endometriosis, etc., and consequently infertility [21][22][23].

Due to the vital importance of reproductive hormones in regulating the development of involved cells such as sertoli cells and granulosa cells, proliferation or apoptosis of ovarian follicles, preparation of ovarian and uterine tissues, supporting fetal growth, etc., several studies have investigated the potential desired effects of ICI on the biosynthesis of these hormones. 

In Eriocheir sinensis, for example, treatment with different doses of ICI resulted in a significant increment in hemolymph estrogen content [24]. 

Moreover, the study by Nie et al. revealed that ICA was able to promote the production of both sex steroids, estrogen, and progesterone, in rat-derived granulosa cells [25]. Similarly, prepubertal administration of ICI, as well as icaritin (ICT), advanced pubertal development in female Sprague-Dawley rats by affecting estrogen and estrogen response gene expression levels [26]. 

POF is an ovarian complication that results in amenorrhea, ovarian failure, and eventually infertility. 

POF is characterized by premature depletion of ovarian follicles in approximately 3% of women younger than 40 years old which is diagnosed by high levels of gonadotropins such as luteinizing hormone (LH) and FSH, and low levels of gonadal hormones including estrogens and inhibins [27][28]. 

Interestingly, the administration of ICI to animal models of D-galactose-induced POF caused a significant reduction in LH and FSH levels, promotion of estrogen and anti-Müllerian hormone (AMH) production, and increased viability of granulosa cells, which were all assumed to be the result of strengthening the DNA repair system indicated by altered levels γH2AX and 53BP1 expression upon treatment with ICI [29]. 

Furthermore, in mice models of D-galactose-induced ovarian aging, in which ovarian aging itself is a causative agent in many ovarian dysfunctions [30][31][32], ICI was capable of inhibiting ovarian follicular atresia, lowering the levels of LH and TSH, improving estrogen biosynthesis and AMH expression, and thereby restoring ovarian function and enhancing fertility in aging mice [33]. 

In addition to the common abnormalities in the secretion of reproductive hormones, there are rare diseases such as perimenopausal depression, which are mediated by an imbalance in the levels of sex steroids, particularly estrogen. 

Interestingly, Cao *et al*. revealed that administration of ICI is followed by a significant increment in the levels of estrogen, testosterone, serotonin, dopamine, and noradrenaline, and lowering the levels of LH and FSH through modulating the phosphatidylinositol 3-kinase (PI3K)/serine-threonine protein kinase (AKT) signaling pathway in a rat model of perimenopausal depression-like [34]. 

Along with PI3K/AKT signaling pathway, ICI is able to affect other signaling pathways in order to modulate reproductive hormones biosynthesis. 

The cyclic adenosine monophosphate (cAMP)/protein kinase A (PKA)/cAMP response element-binding (CREB) protein pathway, for example, is modulated by ICI which is resulted in alteration in estrogen levels and leads to resolving the problem of delayed ovarian maturation [24]. 

Moreover, the CYP17 and CYP19 protein expression, and aromatase levels, are upregulated by ICI that causing elevated steroid hormone secretion in ovarian granulosa cells [25]. 

In addition to the mentioned mechanisms, ICI is able to modulate the expression of hormone receptors in target tissues and thus regulate the effects of reproductive hormones [35].

## Icariin Regulates the Expression of Reproductive Hormones’ Receptors

Although estrogen, the primary female sex hormone, is attributed to be responsible for the regulation of reproductive system function and development of secondary sexual characteristics, it exerts its actions by binding to specific receptors, known as the estrogen receptors (ERs), which in turn activate transcriptional processes and signaling pathways via genomic or non-genomic effects [36]. 

Therefore, any irregularity or dysfunction of these receptors will have the same consequences as the disruption of estrogen levels. A growing body of evidence strongly suggests that the majority of estrogenic actions are mediated by nuclear estrogen receptors, in which two subtypes of ERs, Esr1, known as ERα, and Esr2, known as ERβ, have been generally conserved [37]. Furthermore, the progesterone hormone, which is suggested to be responsible for the human menstrual cycle, pregnancy, and parturition regulation, exerts its function via its nuclear receptors [38] whose activation results in transcriptional regulation [39]. 

Because of the vital importance of steroid hormone receptors in regulating the function of these hormones, a number of studies have examined alterations in the expression of these receptors upon ICI and/or its derivatives administration. In this regard, it is suggested that ICI, and ICT, affected the levels of ERs and PR in both uterus and ovaries which in turn led to the advancement of pubertal development in female rats [26]. 

Moreover, ICI is reported to be capable of regulating the expression levels of ERs in both bone and reproductive tissues in ovariectomized rats suffering from estrogen deficiency [40].

## The Beneficial Effects of Icariin on the Ovarian and Uterine Histoarchitecture

Histomorphology of the major tissues involved in the reproductive process, the ovaries and the uterus, is directly affected by sex steroids through their receptors; Therefore, the physiological structure of these tissues on the one hand indicates the potential fertility and guarantees the effective production of sex steroids and on the other hand, is a manifestation of the suitable functioning of sex hormones and their receptors in these tissues [41][42][43].

The mammalian ovary contains different follicles such as primordial, primary, secondary, antral, and graafian, which are classified based on their stages of development. 

It is well established that the number of primordial follicles is determined during the embryonic period and stays permanent until puberty, referred as an ovarian reserve. In this regard, damage to this ovarian reserve is followed by a pathologic state known as POF or premature ovarian insufficiency (POI). 

Due to the renewal inability of these follicles, depletion of the ovarian reserve could result in permanent infertility [10][44][45]. In this regard, researchers reported that ICI improved the diameter and proportion of ovarian follicles and induced mature follicle development in rodents’ models of aging [33]. In addition, ICI was able to protect the histomorphology of ovarian follicles against an induced state of POF [29]. 

Similarly, ICI alleviated the pathological alterations such as the number of layers of granulosa cells, the mature ovarian follicle, and the corpus luteum caused by perimenopausal depression [34]. 

The promotion of DNA damage repair via altering the expression level of γH2AX and 53BP1, the inhibition of apoptosis through upregulation of Bcl2, and suppression of Bax expression, thus improving the development of ovarian follicles and reducing the number of atretic follicles are the most important mechanisms by which ICI affects the histomorphology of the ovary [25][29][33][34].

In addition to the ovaries, uterine histomorphology is strongly associated with fertility and the possibility of giving birth to a healthy newborn. Thin endometrium, for example, is well documented that is related to a significant decrease in pregnancy rates [46][47]. 

In this regard, Le *et al*. reported that ICI is a promising therapeutic strategy to increase the thickness of the endometrium which exerts its beneficial effects through modulating the expression of VEGF (vascular endothelial growth factors) , CD31, and factor VIII [48].

In ovariectomized animals, which is a model of estrogen depletion, a study suggests that administration of ICI, although it had no effect on uterine tissue weight, increased the thickness of the uterus, endometrial, and luminal epithelium, and the numbers of endometrial glands [40]. 

Furthermore, ICI was able to justify the uterine epithelial heights and myometrium thickness. However, other studies have shown that ICI improves uterine weight in almost identical pathological conditions, too [49][50].

## The Impact of Icariin on the Estrous Cycle and Fertility

In rodents, the secretion of reproductive hormones, the development of ovarian follicles, and the maturation of oocytes are highly regulated processes that occur during a regular cycle, referred as the estrous cycle, that consists of four phases named proestrus, estrus, metestrus, and diestrus. 

This cycle, particularly in rodents like rats and mice, lasts for a 4-5 days period which is characterized to be a repetitive but dynamic process as it exerts different types of cells and levels of hormones [38][51][52]. 

In a study by Kang et al. it was found that ICI administration significantly increased the duration of the estrous cycle in rats which leads to advanced pubertal development [26]. 

The beneficial effects of ICI on the estrous cycle, which was accompanied by alleviated POI, have been attributed to affecting the Nrf2/HO-1/Sirt1 pathway in the mice ovary [53]. More importantly, the administration of ICI to the induced aging led to increased pregnancy rate, improved average litter sizes, elevated average litter birth weight, average weaning weight of litters, and weaning rate revealing the beneficial effects of ICI on female fertility and newborn health [33]. 

ICI beneficial effects on the female reproductive system are shown in Table-1.

**Table:** Table 1.Icariin Exerts Desired Beneficial Effects on the Female Reproductive System

Year	Type	Cell/ Animal	Dose	Duration	Finding	Ref
2020	*In vivo*	*Eriocheir sinensis*	0, 50, 100, and 200 mg/Kg	8 weeks	Icariin was able to increase the levels of vitellogenin, GSI, oocyte volumes, estrogen, and aromatase through the cAMP/PKA/CREB pathway.	[24]
2018	*In vitro*	Rat Ovarian Granolusa Cells	10 μg/l	72 h	ICI caused a dramatic increase in the levels of estrogen and progesterone and the proliferation of granulosa cells. Furthermore, it increased the levels of CYP17 and CYP19.	[25]
2012	*In vivo*	SD rats	20 mg/Kg	20 days	The increased duration of the estrous cycle and increased weight of ovarian tissue resulted after the administration of ICI.	[26]
2019	*In vivo*	C57BL/6 mice	10, 50 and 100 mg/Kg	42 days	ICI improved ovaries weight, ovarian histoarchitecture, decreased LH and FSH levels, upregulated AMH and estrogen, and increased granulosa cell viability through promoting the DNA repair system.	[29]
2018	*In vivo*	Kunming white mice	50, 100, and 200 mg/Kg	30 days	Improved follicular content, reduced apoptosis, decreased LH and FSH levels, increased levels of estrogen, and promoted fertility rates resulted from the administration of ICI.	[33]
2019	*In vivo*	SD rats	12.5, 25, and 50 mg/Kg	30 days	ICI was able to modulate sex-steroid levels as it elevated estrogen and testosterone levels, reduced LH and FSH levels, upregulated ESR1, and decreased apoptosis through PI3K/ AKT pathway.	[34]
2017	*In vivo*	SD rats	50, 100, and 200 mg/Kg	3 respective estrous cycles	ICI could improve the thickness of the thin endometrium via modulating the levels of VEGF, CD31, and factor VIII.	[48]

**LH:** luteinizing hormone, **FSH**: follicle-stimulating hormone. **GSI:** Gonado Somatic Index; **SD:** Sporgue Dawley, **cAMP/PKA/CREB: **Cyclic adenosine monophosphate/protein kinase A/cAMP-responsive element binding protein; **CYP17: **Cytochrome P450 17; **AMH:** Anti-Mullerian Hormone;** PI3K;** Phosphoinositide 3-kinase AKT: Protein kinase B; **VEGF:** Vascular endothelial growth factors; **CD3:**1cluster of differentiation 3.1; **C57BL: **C57 black 6

## Conclusion 

The current literature review demonstrated that ICI could be assumed a novel promising therapeutic strategy in the management of female infertility due to its desired properties such as modulation of the levels of hormones involved in the HPO axis including LH, FSH, estrogen, and progesterone, the regulation of sex-steroid receptors expression in reproductive tissues, preserving of ovarian follicular reserve and the histomorphology of ovarian and uterine tissues, and ultimately the regulation of estrous cycle. 

However, the present studies appeared to be insufficient and the design and conduct of further studies, especially clinical trials, are strongly recommended by the findings of the present study.

## Conflict of Interest

The authors declare that there are no conflicts of interest.
